# Conversion of an oral to nasal intubation in difficult nasal anatomy patients: two case reports

**DOI:** 10.1186/s12871-021-01298-6

**Published:** 2021-03-09

**Authors:** Dong Won Kim, Kyu Nam Kim, Jung Eun Sun, Hyun Jin Lim

**Affiliations:** Department of Anesthesiology and Pain Medicine, Hanyang University Hospital, 222, Wangsimni-ro, Seongdonggu, Seoul, 133-792 Republic of Korea

**Keywords:** Airway management, Intratracheal intubation, Nasotracheal intubation

## Abstract

**Background:**

Nasal intubation is indispensable for some cases that require intraoral surgical access, and the fiberoptic bronchoscope is the best tool for difficult airways. However, fiberoptic bronchoscopy is not always possible in cases with altered pharyngeal anatomy.

**Case presentation:**

In this report, we introduce a novel technique for retrograde endotracheal oral-to-nasal conversion with an ordinary endotracheal tube exchange catheter. A 49-year-old male with a fractured mandible angle and symphysis was scheduled to undergo mandible reconstruction. Secondly, a 45-year-old male who had a bone defect in the mandible angle and ramus was scheduled for mandible and oral cavity reconstruction. We chose to intubate orally first and successfully converted the endotracheal tube from oral to nasal retrogressively using a tube exchange catheter.

**Conclusions:**

Our simple and safe technique, which use a tube exchange catheter retrogressively, provides an alternative method for a difficult airway in which the fiberscope is not helpful.

## Background

Airway management is critical for patient safety, and endotracheal intubation is the most important procedure [1, 2]. The patients in the following two cases had anatomical abnormalities in their faces, and nasotracheal intubation was required to evaluate jaw movement and malocclusion during surgery. Fiberoptic bronchoscopy was not possible due to altered pharyngeal anatomies, and we concluded that direct nasal intubation would be challenging. Therefore, we chose oral intubation and then switched to the nasotracheal tube with a tube exchanger.

## Case presentation

**Fig. 1:**
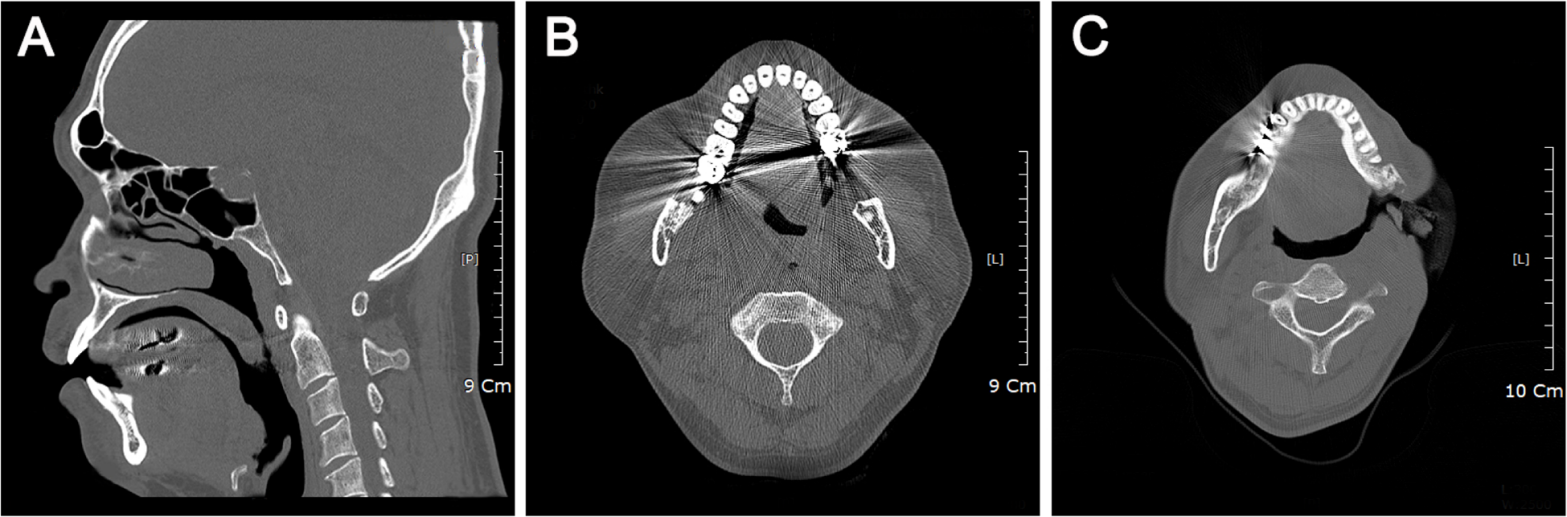
**a** Computed tomography reveals swollen oral mucosa enlargement of the soft palate and uvula in the sagittal plane. **b** Transverse plane. **c** Computed tomography shows bone defect in the left mandible angle and ramus and a skin defect in the left mandibular area with an orocutaneous fistula from complications of radiation therapy following surgery for gingival cancer

A 49-year-old male (159 cm, 71 kg) with a fractured mandible angle and symphysis was scheduled to undergo mandible reconstruction. Although it was difficult to evaluate the Mallampati class, the patient’s neck extension seemed appropriate, and the thyromental distance was more than 3 cm. Upon arrival to the operating room, he remained alert with stable vital signs. Despite appropriate positioning, fiberoptic nasal intubation failed because of swollen oral mucosa around the soft palate and uvula, which obstructed the view of the airway (Fig. 1a, b). Moreover, swollen mucosa interfered with manipulation of the fiberoptic bronchoscope. At this time, effective ventilation was maintained through a face mask. After we proceeded with oral intubation with the McGrath video laryngoscope (Aircraft Medical Ltd., Edinburgh, UK), surgical reduction of the swollen part of the mucosa was performed. We planned to replace the oral tube with a nasal tube.

**Fig. 2:**
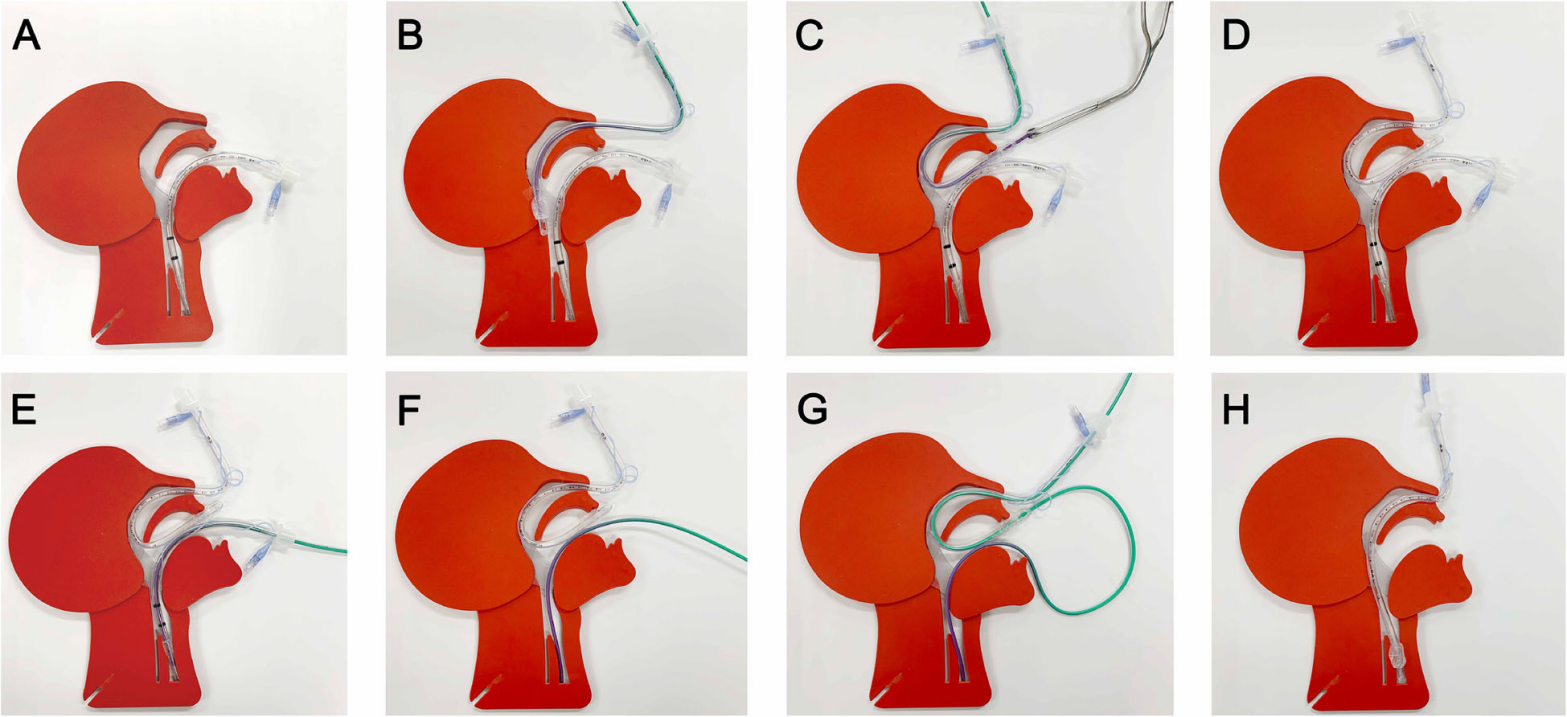
**a** Endotracheal intubation was performed via the oral route for ventilation. **b** The nasal endotracheal tube was inserted through the naris with tube exchanger until it reached the oropharynx. **c** The tip of the tube exchanger was taken out of the mouth with the magill forceps so as to not damage the cuff of the endotracheal tube. **d** The tube exchanger was removed from the nasal endotracheal tube. **e** The tube exchanger was advanced through the orotracheal tube. **f** The orotracheal tube was removed (**g**) The other side of the tube exchanger was inserted retrograde through the tip of the nasotracheal tube, which was pulled out of the mouth. **h** Running along the tube exchanger, the nasotracheal tube was finally advanced into the trachea

First, the left naris was prepared with epinephrine-soaked gauze to prevent bleeding. The nasal endotracheal tube was inserted through the naris with an endotracheal tube exchanger (Cook airway exchange catheter, C-CAE-11.0, Bloomington, IN) until it reached the oropharynx (Fig. 2a, b). Once it appeared in the mouth, we took the tip of the airway exchange catheter out of the mouth with the magill forceps, taking care not to damage the tube cuff (Fig. 2c). After the tube exchanger was removed from the nasal endotracheal tube (Fig. 2d), the tube exchanger was advanced through the orotracheal tube, which remained inside the trachea (Fig. 2e). The balloon of the orotracheal tube was deflated, and we slowly removed the tube while making sure to keep the tube exchanger inside the trachea (Fig. 2f). The outer side of the exchanger was inserted retrograde through the tip of the nasotracheal tube, which had been pulled out of the mouth beforehand (Fig. 2g). Running along the tube exchanger, the nasotracheal tube was finally advanced into the trachea (Fig. 2h). The patient received 100% oxygen during every procedure, and desaturation below 90% was not observed. Total apneic time for the tube exchange (from the time the tube exchanger was advanced through the orotracheal tube until ventilation) was 45 s.

A 45-year-old male (164 cm, 60 kg) with no underlying medical problems was scheduled for mandible and oral cavity reconstruction. He had undergone gingival cancer surgery, including left mandible resection and radiation therapy 8 years previous. Preoperatively, the patient’s airway was reviewed as Mallampati class IV, his mouth opening was about 2 cm, and his neck was somewhat stiff. In the operating room with the patient awake, we used the fiberoptic bronchoscope to explore from the nasal and oral airway to the vocal cords after applying epinephrine-soaked gauze into the nares. The patient had a bone defect in the left mandible angle and ramus and a skin defect in the left mandibular area with an orocutaneous fistula resulting from complications of radiation therapy following surgery for gingival cancer (Fig. 1c). The bone defect in the left mandible made it difficult to enter the glottis with the fiberoptic bronchoscope. Since failed intubation leads to increased risk of epistaxis and swelling of the soft tissue, we performed nasal intubation with the tube exchanger in the manner described above.

## Discussion and conclusions

Nasal intubation was first described in 1902 by Kuhn and is commonly used for anesthesia in oral surgeries when surgical access is needed. Complications of nasotracheal intubation are epistaxis, bacteremia, and partial or complete obstruction of the tube [3, 4]. The most common complication is epistaxis, which can threaten a patient’s life if blood aspirates into the lungs. Abnormal anatomy and numerous intubation attempts increase the risk of complications. In our cases, the patients had a mandible fracture and edematous mucosa in the oral cavity, an orocutaneous fistula, or a facial bone defect. In both cases, nasal fiberoptic intubation was attempted; if continued, the probability of failure and risk of aspiration would only increase. Therefore, we performed orotracheal intubation with video-assisted laryngoscopy.

There are few reported cases that showed exchange of an endotracheal tube from oral to nasal intubation while using the fiberoptic bronchoscope. Dutta et al. reported a case of oral-to-nasal tube conversion with fiberoptic bronchoscopy [5]. They first performed oral intubation using direct laryngoscopy. Then, the bronchoscope was inserted into the naris, and the oral tube connector was cut. The tip of the bronchoscope was inserted through the oral tube to just above the carina, and the fiberscope and tube were gradually withdrawn in a retrograde fashion through the nasal passage. In another case, when the fiberoptic bronchoscope was placed between the deflated tracheal tube cuff and the laryngeal wall, the orotracheal tube was removed, and the nasal endotracheal tube was advanced into the trachea [6].

However, fiberoptic intubation is not always possible due to oral bleeding, secretions, and difficult anatomy. In the present cases, both patients had altered anatomies that obstructed the view. Moreover, it would be difficult to identify the airway on the camera of fiberoptic bronchoscopy due to the postoperative bleeding in the first case. The safe method of tracheal tube exchange ensures airway continuity throughout the procedure and tube exchanger have been designed specifically for this purpose [7].

There are similar cases reporting endotracheal tube exchange using a tube exchanger. In one case [8], oral bleeding complicated fiberoptic bronchoscopy, and they used the tube exchanger consisted of two parts that could be separated or firmly connected end-to-end. Another case introduced oral-to-nasal tube exchange using a newly designed endotracheal tube exchanger [9]. This special tube exchanger could be separated into two parts, whereas our technique only requires a basic airway exchanger. In addition, the fiberoptic intubation method under general anesthesia does not always provide enough oxygen during the procedure. Because our method is using an already secured airway and there is no need to secure visibility for direct nasal intubation using fiberoptic bronchoscopy, it provides a shorter non-ventilating period.

A drawback of this method is that it can be safely applied to a stable patient whose functional residual capacity is preserved. Considering the situation in which the tube exchange is difficult, sufficient preoxygenation and preparation for re-attempt of oral intubation should be required.

In conclusion, although it might not be the first choice for nasotracheal intubation in difficult airways, our simple technique provides an alternative method in which the fiberscope is not helpful. It can be applied without proficient manipulation of the fiberscope.

## Data Availability

The datasets used and analysed during the current study are available from the corresponding author on reasonable request.

## References

[1] Hews J, El-Boghdadly K, Ahmad I (2019). Difficult airway management for the anaesthetist. Br J Hosp Med (London, England: 2005).

[2] Frerk C, Mitchell VS, McNarry AF, Mendonca C, Bhagrath R, Patel A, O'Sullivan EP, Woodall NM, Ahmad I (2015). Difficult airway society 2015 guidelines for management of unanticipated difficult intubation in adults. Br J Anaesth.

[3] Hall CE, Shutt LE (2003). Nasotracheal intubation for head and neck surgery. Anaesthesia.

[4] Prasanna D, Bhat S (2014). Nasotracheal intubation: an Overview. J Maxillofac Oral Surg.

[5] Dutta A, Chari P, Mohan RA, Manhas Y (2002). Oral to nasal endotracheal tube exchange in a difficult airway: a novel method. Anesthesiology.

[6] Wolpert A, Goto H (2006). Exchanging an endotracheal tube from oral to nasal intubation during continuous ventilation. Anesth Analg.

[7] Higgs A, McGrath BA, Goddard C, Rangasami J, Suntharalingam G, Gale R, Cook TM (2018). Guidelines for the management of tracheal intubation in critically ill adults. Br J Anaesth.

[8] Nakata Y, Niimi Y (1995). Oral-to-nasal endotracheal tube exchange in patients with bleeding esophageal varices. Anesthesiology.

[9] Salibian H, Jain S, Gabriel D, Azocar RJ (2002). Conversion of an oral to nasal orotracheal intubation using an endotracheal tube exchanger. Anesth Analg.

